# Quarantined, Sequestered, Closed: Theorising Academic Bodies Under Covid-19 Lockdown

**DOI:** 10.1007/s42438-020-00193-6

**Published:** 2020-10-01

**Authors:** Lesley Gourlay

**Affiliations:** University College London Institute of Education, London, England UK

**Keywords:** Quarantine, Covid-19, Sociomateriality, Embodied practices, Online teaching

## Abstract

The term ‘quarantine’ is derived from the Italian *quarantena*, from *quaranta*, referring to the forty days of isolation traditionally imposed during the era of the Black Death in Europe. This paper examines this and related contemporary terms, in order to consider the complex and contradictory nature of enforced sites of isolation, with reference to the historical literature. The centrality of spatial practices in the current pandemic is emphasised, with a focus on the normally unobserved, micro practices of individuals under ‘lockdown’. The paper reports on an interview study conducted at a large UK Higher Education institution during the Covid-19 ‘lockdown’, and analyses the accounts of six academics, focusing particularly on their embodied and sociomaterial practices, with reference to the etymological analysis. The paper considers the extent to which their reported experiences reflect the various meanings of the term *sequestrato*, going on to propose that their working practices, particularly focused on screens and video calls, are characterised by a need to ‘perform the university’. I speculate on how the ontological nature of the university itself has been fundamentally altered by the closure of the campus and lockdown, proposing that the site of the university is now radically dispersed across these sequestered bodies. I conclude by calling into the question the accuracy of the term ‘online teaching and learning’, instead suggesting that in a fundamental sense, none of these practices is in fact ‘online’ or digital.

## Introduction

At the time of writing, countries around the world are emerging from ‘lockdown’ arrangements which were put in place in order to contain the spread of the 2020 coronavirus and the resultant illness, Covid-19, by asking populations to remain in their homes, only leaving for essential purposes. These arrangements vary across national and regional contexts, and are ongoing in many locations due to continued outbreaks of the virus. ‘Lockdown’ has required millions of people to work at home where possible, and educational institutions were forced to close at short notice. This paper focuses on an interview study which investigated the impact of the lockdown on academics at a large UK university, exploring their accounts from two theoretical perspectives. First, I consider the etymology of some of the key terms associated with the historical concepts and practices relating to ‘quarantine’, in particularly the tensions inherent in the terminology, and how these are demonstrated in the contemporary accounts of these UK-based academics under lockdown. I then go on to focus on the material and embodied practices engaged in by the interviewees, in order to engage in digitally mediated communication. With reference to interview data and also images supplied by the interviewees, I argue that these practices—while framed as ‘digital’—are in fact fundamentally material. I conclude by proposing the screen as a ‘portal’ for the performance of academic and professional subjectivities.

### *Quarantena*

The term ‘quarantine’ is derived from the Italian *quarantena*, and was originally used to refer to a period of forty (*quaranta*) days of confinement. As Bashford explains in her history of quarantine:

In medieval and early modern Europe, it was employed or strategies of purification broadly conceived, from Lent to the lying in after childbirth. In the face of plague, as in these contexts, the 40-day period was intended as a prophylactic and therapeutic measure, used to provide comfort and meet spiritual and practical needs (including basic food, water, medicines, and clean, warm accommodation) thereby healing both the body and the soul of patients… As the fixed-term, spatial separation of patients, underpinned by concern about contagion, quarantine was a specific form public health within the broader culture of chronic institutional care for social, medical and moral concerns which developed in medieval and early modern Europe.

(Bashford 2016: 16)

Bashford alludes to terms which were used at that time in the Mediterranean Basin to describe those who were placed in *quarantena*, they were said to be *sequestrato* or *serrato* (Bashford 2016: 16). In this section, I will examine the etymology of these words, and their modern English translations, in order to explore the nature of our contemporary *quarantena*, the coronavirus lockdown. The etymology of the verb ‘to sequester’ reveals that it came from the Middle English *sequestren*, which was derived from the Anglo-French *sequestrer*, which came from the Latin *sequestrare*, meaning ‘to hand over to a trustee’ (Merriam Webster Online Dictionary 2020). It is defined by Merriam’s as (1a) too set apart, segregate, (1b) to seclude, withdraw, (2a) to seize especially by writ of sequestration, and (2b) to place (property) in custody especially in sequestration. The synonyms are given as cut off, insulate, isolate, seclude, segregate, and separate, and its antonyms are desegregate, integrate, and reintegrate. Dictionary.com defines to sequester as follows:

1. To remove or withdraw into solitude or retirement; seclude
2. To remove or separate; banish; exile
3. To keep apart from others; segregate or isolate
4. Law: to remove (property) temporarily from the possession of the owner; seize and hold, as the property and income of a debtor, until legal claims are satisfied.

Dictionary.com also gives a slightly different account of the etymology of the word, coming from Old French *sequestrer* from late Latin *sequestrare* ‘to give to someone to keep safe’, which came from Latin *sequester* ‘someone given something to keep safe’. The Collins online Italian-English dictionary gives the translation of *serrato* (the past participle of the verb *serrare*) as (1) locked, (2) dense, compact, crowded, and (3) clenched, clasped/clamped, tightened. The Italian online dictionary Trecanni (2020) provides two definitions in Italian, (1) *chiuso*, and (2) *stretto*, *ristretto*. Collins translates *chuiso* into English as shut, closed, or locked. It translates *stretto* as narrow or tight. The word can also mean close, strict, or exact. *Ristretto* is translated as limited, restricted, narrow, closed, or hemmed in.

An exploration of these terms associated by Bashford with the medieval *quarantena* reveal a complex web of meanings. The notion of restriction is consistent throughout, but in the case of Merriam and also the Dictionary.com definitions of ‘sequester’, there are both positive and negative senses of the word. It can mean a withdrawal into solitude and seclusion, which suggests a sense of self-protection, or even a healing process, and has positive connotations. However, the word also carries meanings of banishment, exile, and isolation. I will return to this contrast later in the paper in my discussion of the interview data.

## Spatial Understandings of the Current Crisis

In a paper referring directing to Covid-19, Brinks and Ibert (2020) explore the spatial dimensions of the current crisis. They propose ‘…a conceptual shift from the structural conditions that cause crises to an actor-centric approach focused on the practical consequences of crisis of individual and collective agency’ (Brinks and Ibert 2020: 276). They argue that the crisis management literature is dominated by temporal aspects, and ‘…lacks a systemic approach to integrate spatial imaginations into theories and practices of crisis management’ (Brinks and Ibert 2020: 276). They set out the defining features of a crisis, which are *uncertainty*, *urgency*, and *existential threat*. They also highlight the emergent nature of a crisis, and the fact that at the onset of the crisis is characterised by uncertainty about both the present and the future:

Under such conditions, action does not take place within a given frame of meaning. Rather, in crisis participants are forced to learn by interpreting the situation tentatively while acting on it. Therefore, crises are usually perceived twice. In a first loop, participants encounter a critical turn in the course of events surprisingly. They experience an open-ended phase of chaos and escalation during which they struggle to regain control while action and sensemaking remain incompletely connected.

(Brinks and Ibert 2020: 278)

They go on to describe a second loop of crisis experience when the participants move into a ‘new normal’. They define the coronavirus as a ‘transboundary crisis’ (Boin and Rhinard 2008) that affects all geographical areas and elements of society. However, they argue that understanding of spatiality of the crisis has been underdeveloped. They propose the TPSN framework developed by Jessop, Brenner, and Jones (2008). This consists of *territory*, *place*, *scale*, and *network*, and goes on to provide examples relating to the current Covid-19 crisis. Under *territory*, they give the example of the portrayal of the outbreak according to territorial entities, such as the depiction of the spread on world maps. An example of *place* is the emergence of specific locations, such as epicentres or locations of ‘super-spreader’ events. *Scale* is exemplified by the assignment of responsibility, such as to the World Health Organization. The example of *network* they give is of expert communities. Jessop, Brenner, and Jones (2008) do not regard these as clearly separate categories but rather as interwoven together in ‘sociospatial relations’ (Brinks and Ibert 2020: 281); they go on to discuss three nexuses of these.

The first they examine is *network-place*, which is of most relevance to this paper. They illustrate this with reference to how places like supermarkets have been physically rearranged for increased safety from the virus, with the addition of Perspex panels and other measures to assure social distancing. They also point out the networked status of each supermarket as part of a chain, and therefore part of a *network of practices* (Brown and Duguid 2001). Their analysis is helpful in setting out a framework which can provide some theoretical purchase on the crisis in terms of spatial and material practices; however, the individual domestic settings of individuals under ‘lockdown’ defy analysis using the TPSN framework. The *network-place* nexus cannot apply, given the lack of collective agreement on spatial arrangements in individual people’s homes, and the distributed and idiosyncratic nature of these arrangements. Instead, arguably, these spatial practices remain unobserved and fragmented, and undertheorised in our understandings of the nature of the contemporary lockdown, and digital practices within it. A more granular analysis is needed, which can take into account the nonstandardised, improvised, and emergent nature of these sociospatial relations.

In an edited historical collection focusing on practices of isolation and exclusion in the modern world, Strange and Bashford (2003) point out the spatialised nature of both correctionalism and medical isolation:

While medical and penal historiography rarely intersect, historical practices of correctionalism within prisons and the punitiveness of medical isolation in modern democracies are difficult to distinguish. Public health, itself a modern enterprise loosely tied to governance of populations was, and in certain contexts still is, an inherently spatialised set of practices. From early quarantine to the eighteenth century ‘medical police’ to the health regulation of immigrants at national borders over the twentieth century, the medical and penal have dovetailed as tactics to define and manage problem populations as spatial strategies involving precise geographies of isolation, similar imperatives towards internal segregation, and shared histories of the policing of boundaries of exile and enclosure.

(Strange and Bashford 2003: 3–4)

They go on to explore the tensions between those being confined as being under protection and vulnerable, while also being regarded as a danger. With reference to Castel (1991), they point out the move at that time towards confinement strategies in the late twentieth century in which ‘…the preventative-punitive rationale slid into the “therapeutic” and even into the “restorative”’ (Strange and Bashford 2003: 6). Bashford (2003) in the same volume examines how ‘… in concert with preventative segregation, public health detention has also involved installing habits and rules of self-governance into the bodies and the souls of the “dangerous”’ (Strange and Bashford 2003: 6).

With reference to sites of isolation such as leper colonies, they discuss the role of place-making as, ‘…the rendering of certain spaces into undesirable zones of exclusion, or into enclosed sites of confinement and incarceration’ (Strange and Bashford 2003). They explore how sites of isolation can derive their meaning as a result of their physical features, such as islands, while in contrast ‘…exclusionary practices impose new meanings on space by design. The erection of buildings, compounds and barriers provides visible evidence of exclusion but within those sites elaborate internal systems of partitioning produce interior forms of segregation’ (Strange and Bashford 2003: 9). They consider the role of walls, in particular the ‘ha ha wall’, which was a feature of the design of mental asylums. The ha-ha was a retaining wall built at the bottom of a slope of land, and so was visible from the outside as an imposing structure of confinement, but was invisible to the insiders, who were provided with an illusion of freedom. Bashford and Strange consider the symbolic meanings of the ha-ha wall, which ‘…offered both protection *of* and protection *from* certain populations; it expressed different objectives simultaneously, depending on one’s position relative to it, as heterotopic carceral and segregative practices always do’ (Strange and Bashford 2003: 10). Looking at the subjectivities of those who are confined, they point out the reflexive relationship between place and identity. As they put it:

All exclusionary practices put individuals and collectivities ‘out of place’ but modern forms of exclusion aimed further to put people *in* their place in order to protect, cure or reform. The constant spatial and temporal surveillance in places of isolation, combined with intervention over the minutest aspects of daily life, has been driven by the desire to produce desirable subjectivities.

(Strange and Bashford 2003: 12)

They refer to Foucault’s ‘techniques of the self’ and the cultivation of isolated selves. Their collection examines the interior lives of people forced into exclusion, and the ways in which spaces of isolation are reshaped by those within them.

Clearly, Strange and Bashford are focused on formal institutions of isolation, such as prisons and leper colonies, as opposed to peoples’ homes, in the situation of the current Covid-19 lockdown. The ‘inmates’ are not there to be punished, but to be protected and to protect others from the virus. Unlike the contexts they discuss, there is no sense of the isolated being ‘the other’, in a scenario in which the whole population is isolated. So, the lockdown differs from these historical sites of isolation in various important respects. However, there are—I would argue—several strands of analysis set out above that are relevant to the present consideration of academics in lockdown, and that echo the tensions inherent the notion of being *sequestrato*, as discussed above. The tension between protection and seclusion, versus forced and unwanted incarceration is one, and the process by which individuals under lockdown shape their subjectivities through embodied practices in interaction with spaces and material practices is another. There has been a growing awareness of the importance of sociomaterial perspectives in education in recent years (e.g. Fenwick, Edwards, and Sawchuk 2011) and also of the role of spatiality in education (e.g. Acton 2017; Bayne, Gallagher, and Lamb 2014; Enriquez 2011; 2012). This paper will adopt this perspective in the analysis of the interviewees’ responses to the lockdown.

In her consideration of the social definition of the current coronavirus pandemic, Pfister (2020) points out the way in which the pandemic appears:

The social definition of the pandemic becomes obvious through the fact that for the major part of the public—unless one is caught up in the midst of the breakdown of the healthcare system—the pandemic appears through the daily checking of epidemiological maps, the prevalence of the virus (the affected part of a given community), incidences (new cases for a given period of time), and rising death tolls. Unlike most other disasters, the pandemic lacks direct evidence. Therefore, it is built on an assemblage of information, and hence is situated in the sphere of knowledge.

(Pfister 2020)

With reference to Strong (1990), she points out three psychological epidemics which accompany a microbial epidemic. These are firstly fear and suspicion, secondly explanation and collective disorientation, and thirdly what she calls an ‘epidemic of action’, which she argues, in the case of coronavirus, consists of the disruption of routines. As she puts it ‘[p]aradoxically, the suspension of order is considered the means of salvaging it’. This paradox again seems to echo the tensions inherent in the *quarantena* predicament as discussed above.

## Academics’ Experience of Lockdown

In March and April of 2020, Nurse and O’Neill (2020) explored academics’ experiences of the current Covid-19 lockdown using a walking and imagining exercise, in collaboration with four other authors. The instructions to the researchers were to write about either (a) their memories of walking and imagining in one of the rooms of your house/apartment/while exercising during the last two-three weeks, or (b) undertake a slow walk in your apartment, flat/house moving from room to room. Stop at a place, a photograph or an object that calls you to pay attention. They asked, why does this have resonance for you? What does it evoke and invoke biographically? Four main themes emerged. The first of these was ‘daily routine now and then’, which they comment on as follows:

Daily routine has changed for us all, as have the spaces and pace at which we now live. As researchers we reclaim our homes, and create liveable that collaboratively (re)connect our present with our past through our memories. (Nurse and O’Neill 2020)

The participants in the study describe how they have reconfigured their living spaces in order to make them into workplaces. They comment that they are now full of ‘un-office’ like images and sounds. Katarzyna comments:

Isolated with my husband and two teenage children, surrounded by pictures of our ancestors we were constantly kindly asked and harshly ordered to reorganise our everyday routines, to transform our place into lecture room, into classroom and engineering office. [Katarzyna]

Lyudmila reflects on how her house has changed:

The whole house changed shape and some familiar and accessible areas became ‘offices’: on-line, virtual meeting places; on-line teaching music zones; violin practice rooms. All members of my family continued to work, to walk, to practice musical instrument and to exercise, but at their own paces. [Lyudmila]

They also identify the theme of ‘walking through our memories’, referring to a strange ‘taste’ of silence, and a sense of distance and space:

Everything now looked almost surreal and distant, like boarding passes, concerts tickets and programmes. I looked at those trying to recollect in my memory happy occasions. Then journals. Sociology journals were piling around for a while, as I tried to find a spare minute to read them. Now is the time. There is a certain pleasure of reading article after article and to enjoy reading and thinking... [Lyudmila]

They also consider what they call ‘the autobiographical meaning of Covid-19’, with the participants speculating on how they will look back on the lockdown. Lyudmila reflects:

What are the sounds of isolation? The first thing which might immediately come to mind is silence. Silence could be imagined as a natural faculty of isolation. And its synonyms include separation, segregation and yes, very familiar to us quarantine. However, the experience of recent self-isolation with an entire family brings to life different shades of sounds of self-isolation and memories. [Lyudmila]

This piece captures some of the complexities of our contemporary *quarantena* for these academics but is a very short piece which does not go into detail about these academics’ perspectives. In the next section, I will report on an in-depth interview study exploring the experiences of academics under lockdown, in order to gain insights into the particular nature of the contemporary Covid-19 lockdown.

## Interview Study

The UK lockdown in response to the coronavirus crisis commenced in March 2020. At University College London Institute of Education, the ‘Moving to Teaching Online and Homeworking’ study was initiated shortly afterwards by Professor Allison Littlejohn (UCL IOE 2020) to explore the impact of this sudden move to working online on UCL staff. Ethics clearance was granted, a survey was administered to all UCL staff, and at the time of writing, almost 500 responses had been received. The survey focused on their experiences of lockdown and their attitudes towards it, and also asked participants to supply a photo or other images to represent their experience. Survey respondents were also asked if they would be willing to be interviewed, and over 120 volunteered. Thirty of these volunteers were selected to be interviewed; these were chosen in order to provide a representative cross section of the staff in terms of gender, age, seniority, role, ethnicity, and sexuality. The interviews were semi-structured, with the questions and themes focused on the participant’s experience of the beginning of the lockdown, and how they had adapted in terms of their practices, spatial and sociomaterial networks which they had set up in their domestic spaces in order to engage remotely using digital devices. The interviews included a focus on their ‘typical lockdown day’, and also explored how the lockdown had affected the nature of the work they were engaged in, any challenges they had faced, and how they felt it had impacted their teaching and also research work and contact with their academic communities at the university and beyond. The perceived impact on their students was also explored, and how their relationship and engagement with them had been affected. There was also discussion of the practical and emotional impacts of the lockdown, revealing a range of feelings, both positive and negative, plus discussion of challenges of family life and childcare. The qualitative comments provided by the participants in the survey were reviewed and discussed in more depth, and if the participants had provided an image as part of the survey, interviewees were also invited to discuss the image they had provided and what it represented to them. The interviews were divided up and conducted by four researchers across the team. This paper refers to a subset of the data, focusing on six of the twelve members of academic staff I interviewed personally. These were selected for analysis as their accounts and vignettes were particularly salient to the theme of this paper, in terms of their spatial and sociomaterial practices.

The interviews were conducted on Skype, then professionally transcribed. Participants were provided with the transcripts and had the opportunity to ‘member check’ them, making any redactions they felt necessary in order to preserve their anonymity and that of colleagues, students, or departments. Pseudonyms were used throughout, and any identifying features were removed from the transcript by a further checking process within the research team. The transcripts were then uploaded into NVivo and thematically coded. This paper presents an analysis conducted by close reading of comments related to images provided by these six participants, focused particularly on the themes of spatial and sociomaterial practices, in addition to comments relating to the aspects of the state of being *sequestrato*, as discussed above.

### Nuala’s Icons

**Fig. 1:**
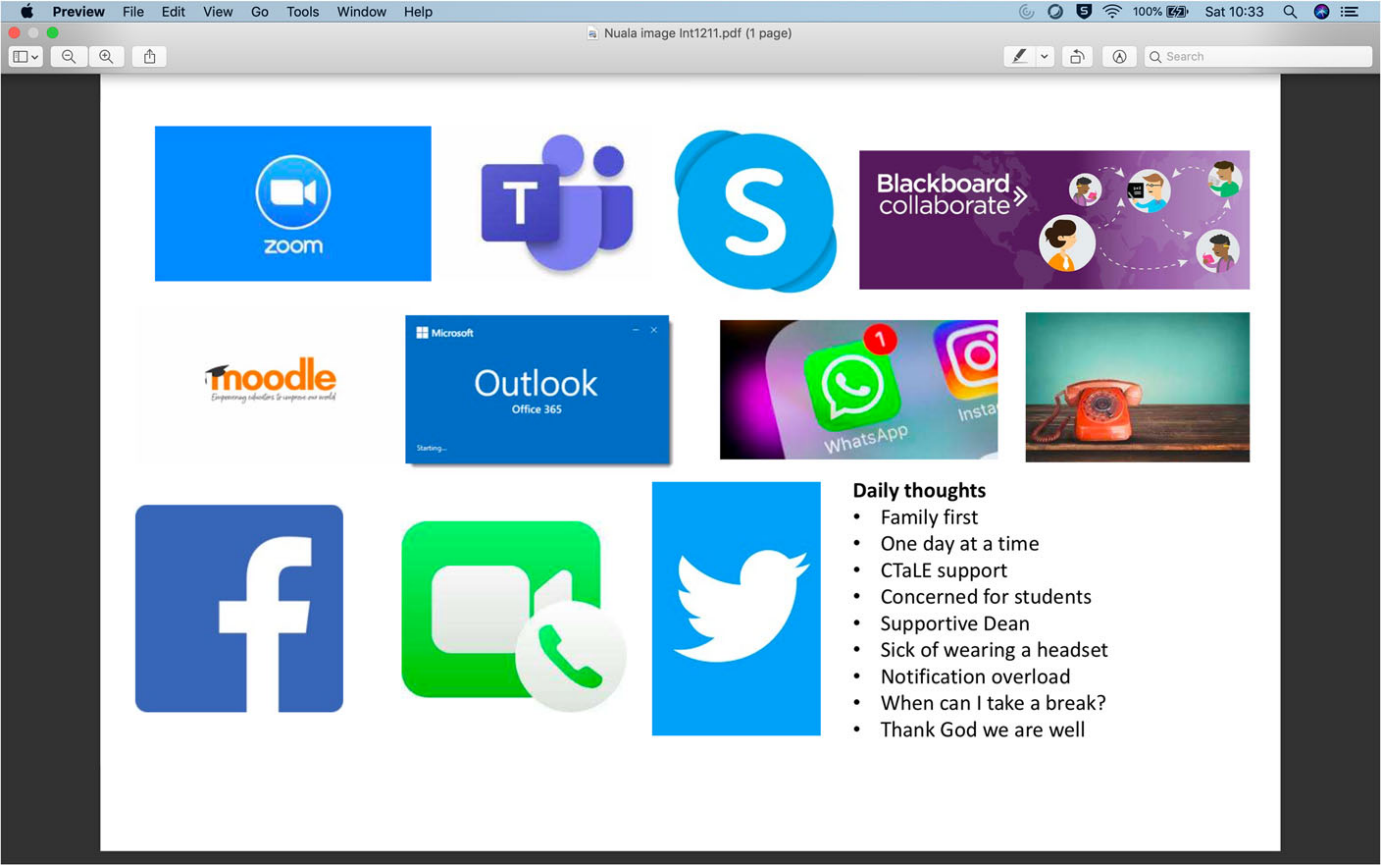
Image provided by Nuala

Nuala is a Teaching Fellow in a large department and has programme leadership responsibility. She came into academia from her previous profession. She provided this image (Fig. 1) with her survey.

Nuala created the image above to represent her experience. It is composed of a series of icons of different online platforms, plus a photo of a telephone. It is noteworthy that all are concerned with communication, except the icons for Blackboard Collaborate and Moodle, which are used for online teaching. Her list of ‘daily thoughts’ reveals some of the challenges she was facing at that time. She alludes to the need to care for her family. She uses the expression ‘one day at a time’, suggesting emotional difficulty. She also expresses concern for her students, and gratitude that her dean is supportive. She also states she is ‘sick of wearing a headset’ and has a sense of ‘notification overload’, which echoes the focus on communication technology in her images. Her question ‘when can I take a break?’ implies fatigue and overwork. In her interview, she refers to this sense of being overwhelmed by technology, with ‘everything binging’:

Um, now, we have run lots of seminars about education. So, we’ve kind of done stuff ourselves, to build that. Um, and I feel that you know, there’s Teams binging, there’s emails binging, there’s everything’s binging. Um, so there was a point where… I think one of the things was because the start of it was the Easter holiday.

She tells me she had been off sick for a time before the lockdown and had been going to the campus just a couple of days a week due to her need to recover. She describes how work has been under lockdown. Despite an increased workload, for Nuala, the lockdown has led to an improvement in her chronic health condition, due to not having to commute:

So, it kind of feels like yeah, I’ve been really busy. Um, busier than I was supposed to be but because I’m not commuting and I’m not going in, my health has been much better than it had been.

A lot of her interview focuses on her attempts to understand the guidance given by the university at that time about what the students might expect from the next academic year, and her challenges around what she should tell the many students making enquiries about whether there would be face-to-face teaching or not. She describes how she was advised to strictly use a particular phrase which was being used by the Marketing department, when communicating with the students, while behind the scenes she felt there was a lot of confusion. Nuala does not directly allude to her sociomaterial setup, but her account reveals a sense of digital overload and being overwhelmed by the range of different technologies she was engaging with under lockdown.

### Courtney’s ‘Toadspace’

Courtney is a Postgraduate Teaching Assistant, whose contract had come to an end at the time of her interview. She explained to me that she is only employed by the university during term-time, and has to do ‘gig economy’ work during the summer, such as working at festivals, which had all been cancelled this year. She explained that she was living on her savings over the summer and would need to reapply for her job. She has also been unable to respond to research calls over the summer due to her unemployed status. However, despite these hardships, she stated that the lockdown had not been an entirely negative experience for her:

I love… However, I love working from home and being around my housemates, some of my best friends. So, it sounds like it was a sort of really, kind of, mixed or complex experience.

Later in the interview, she alludes to the fact that there have been no job vacancies during lockdown, but also that not having to constantly make applications has been ‘…a wonderful rest’. She talks more about the positives she has found in the experience:

I don’t know. Like, I mean, I think the, the, the thing that I’m starting to tell people now is, like, how much of a positive experience I’ve had in lockdown. At the beginning, when everyone was really struggling, it felt very faux pas to be, like, well, I’m having a great time. Um, uh, just because, you know, I have, like…Fixed, fixed my sleeping, I’ve got a good, good set of routines, um… Like, I mean, it, it’s an extremely privileged position to have, that, like, a lot of my jobs are cancelled, but I can still keep doing some of them.

**Fig. 2:**
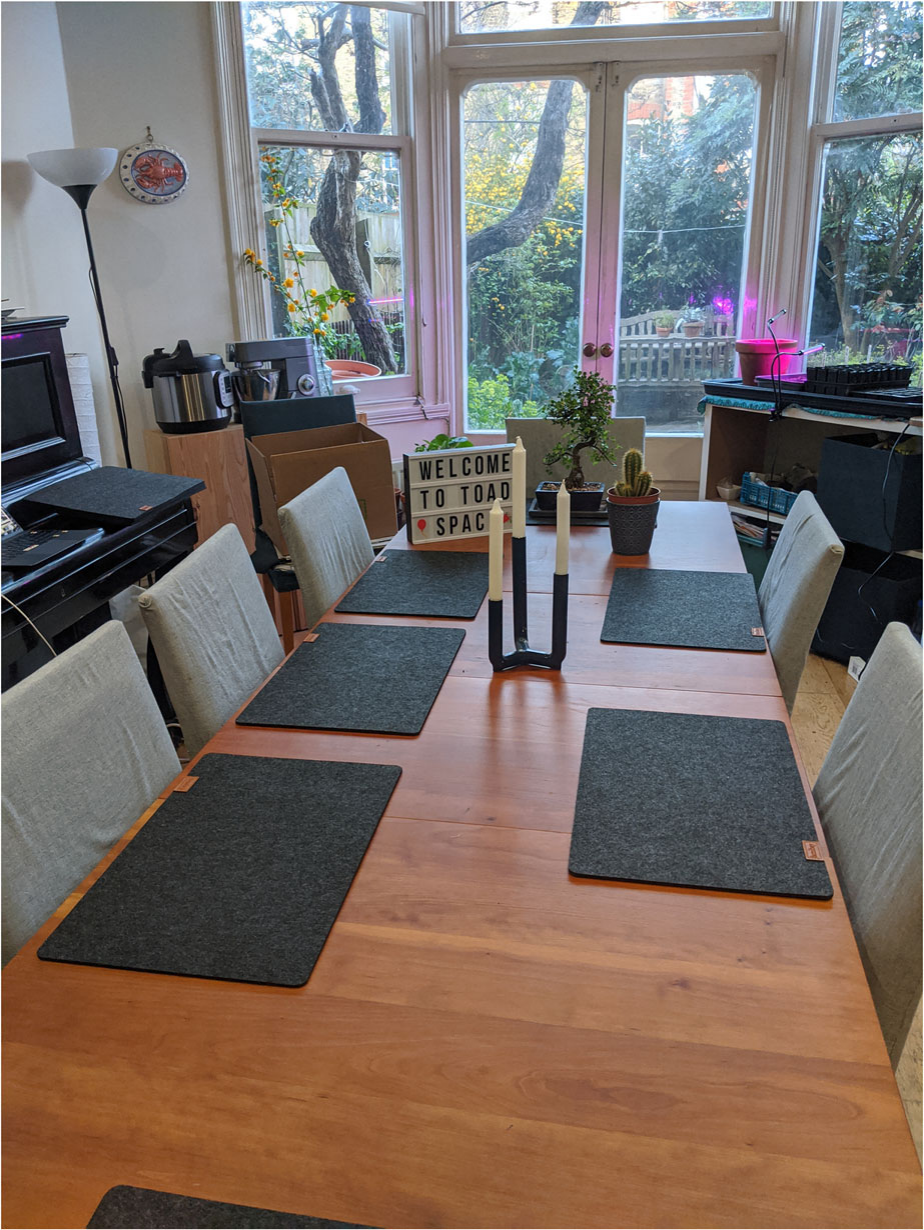
Image provided by Courtney

She provided this image (Fig. 2) of a large table, with the label ‘Toadspace’.

In her interview, I asked her about it and she explained that she lives in a large shared house with six other flatmates all working at home, and that one of her flatmates who is a graphic designer had arranged the room for them to be able to have work space available. They are a very close group of friends, who give themselves the nickname of ‘the toads’. By rearranging this space and naming it, Courtney and her flatmates have reconfigured their space and spatial practices in response to lockdown. In her interview, Courtney also shows me a curtain she has rigged up against a bookcase in her bedroom, which she uses as a backdrop for her work-related video calls. She describes how she regards it as her ‘professional’ space, rather than have her personal possessions and space appear in the background. She stated:

I actually got to, got to a point where, uh, because I’m, I guess, also trying to market myself as, like a science communicator, uh…And I’ve done a lot of public lecturing over the past year. Uh, I do a lot of public events and, starting in late March, I decided to do online lectures, um, which, which have been really, really great. Everyone seems to really enjoy those. Uh, um, I was, like, I, I don’t… I think I need to disambiguate my public persona from my private persona. It’s getting, like, all a bit muddled.

She also related how she has a medical poster which she uses as a backdrop for online lectures. In both cases, she is staging a physical space within the intimate setting of her bedroom and designating it as ‘professional’.

### Ellie’s Bedside Desk

**Fig. 3:**
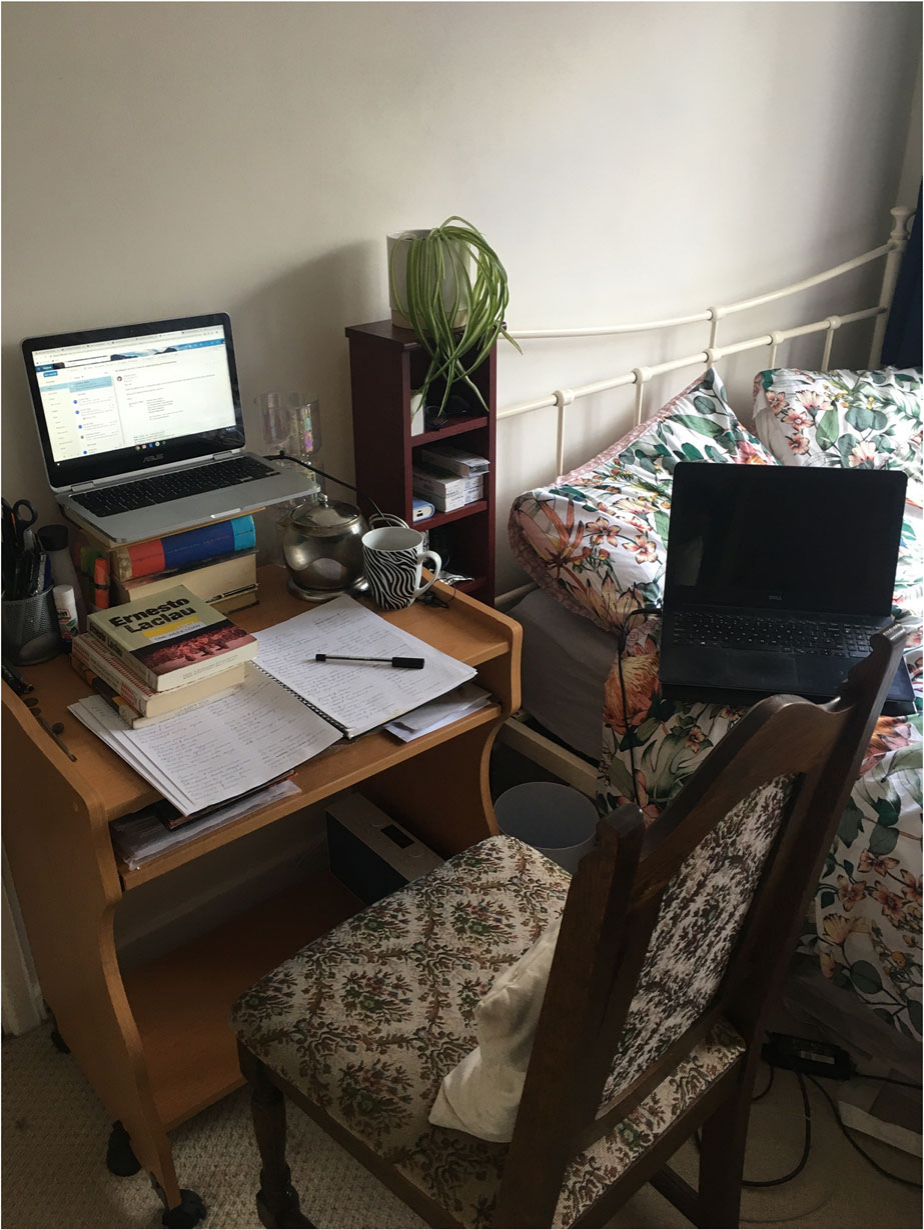
Image provided by Ellie

Ellie is a Masters graduate and a Research Assistant in a specialist research unit. She told me in her interview that she had been sharing a flat in London when the lockdown started. Her work space in the flat was her small bedside table, as shown in Fig. 3.

I think my, I mean my day was very um very much um, I’d, I’d get up and have a shower and then go back to the room and sit at that table and start work. Um and I think the experience, it was very hard to get that sort of mental divide between, um, between work and home um and that um, that makes it harder to switch off at the end of the day but it also makes it a lot harder to sort of switch on. Um, you don’t get that feeling of walking into the office and um going into work mode and focusing and knowing what you’re doing. Um, so it was, it was quite difficult in that sense. Um obviously it’s, it’s, hard just not having enough physical space to work in. Um, I, I, I am spending a lot of time sort of juggling, saying okay if my keyboard goes here where can I put my notepad? But I need to make notes, but I’ve got to have the keyboard and yes, it’s, it’s just a constant minor irritation.

As with Courtney, Ellie has reconfigured her space. In this case, she had to create a work space in her bedroom, as there was no communal space available to her. The assemblage she created was fragile and difficult to maintain, with inadequate room for the artefacts she needed and a constant need for ‘juggling’. Ellie was forced to leave the flat in London as her flatmates were freelance workers, lost all their work, and could not pay their rent. She moved to her parents’ house two hours outside London. In her interview, she describes how isolated she feels, being away from her friends and workmates.

### Sophia’s Virus Lock

Sophia is a Senior Lecturer from southern Europe. In her interview, she describes leaving London on one of the last flights to her home country, an experience she describes as ‘traumatic’:

So, so I stayed home for a week before, um, I decided that perhaps it was better to actually move to here… So, on 23rd March I took one of the very last flights… Over to here which was… I count that as a traumatic experience, to be honest, because obviously there was such a high risk of getting it on the plane. Ah, I was in constant communication here with my family and they were saying, you know, this plane came from the UK and there were four cases and now they’re in the hospital, so… But I took, I took the risk and, um, so I experienced it as a… Um, so, so, first I got informed and then when I realised that, that this was going to get out of hand in the UK, I, I had this feeling of then, of urgency that I just need to get out. So, I feel that now I understand how refugees feel because I got that feeling of there is a place that perhaps I’ll be safer.

She did not contribute an image when she first filled out the survey, so I asked her what image she would use to represent her experience. She replied that it would be ‘a lock in the shape of a virus’. I asked her to explain:

So, it would be the virus and then in the middle there would be the, the hole for the, the key. Um, and it would be like that because, ah, this whole situation created a boundary for us in everything that we do in our lives and, ah, around that boundary we, we created new dynamics and we created new, ah, experiences. So, things that were not planned happened urgently. Then, ah, things that were planned did not happen, they were cancelled. Ah, we had time I think for ourselves to sit and think as humanity, all of us, and I think that’s the very positive thing. But I, I read on Facebook somebody wrote, ah, I feel like I’ve been naughty and I’ve been sent to my room, and I said, you know what, [laughs] that is exactly what is needed perhaps. Ah, so, so there is, there is that boundary where, ah, we, we… There was more thought. There was less excess which I think is, is key in, ah… In business, excess is the worst possible thing. Business is all about efficiency, but, ah, our lifestyle has evolved into something which is completely excessive.

Sophia’s experience was one of flight to safety and exile, but interestingly she also mentions the notion of punishment and incarceration in her quote above about the virus as a lock.

### Lawrence’s Tower of Tables

Lawrence is a lecturer who also practices in his profession part-time. His interview focuses on the sudden move to online teaching and the impact on his students. He also discussed the loss of access to historical archives for his research, and also the cancellation of research-related trips and the subsequent loss of contact with his wider academic community:

Right now, there’s probably enough material that I, that I have, and I… But as I said, it’s not sustainable because that, that will dry up…Um, and the access to those sources, that I can’t get, it will, it will eventually dry up. I mean, I was meant, as I said, I was meant to go to (names country) for a research trip. In April, for three weeks. Um, so that was cancelled, and I don’t see that coming back. Um, that’s something… You know, that’s, that’s way beyond UCLs, um, problem… Um, I accept that. So, so yeah, just the, the general archives, I suppose, in, you know, (names cultural institution) or the archive collections that I would rely on at (names cultural institution), um, just within London. But a lot of my work is with, I suppose, partnerships within Europe or, possibly, in, in China and in Africa. Um, there are a couple of projects that I’m working with in (names country), and in (names country), which, I was due to travel there…Next week, and then also in July. So those, obviously, aren’t happening. Um, so those smaller trips, where one would expect to, you know, to build professional relations.

**Fig. 4:**
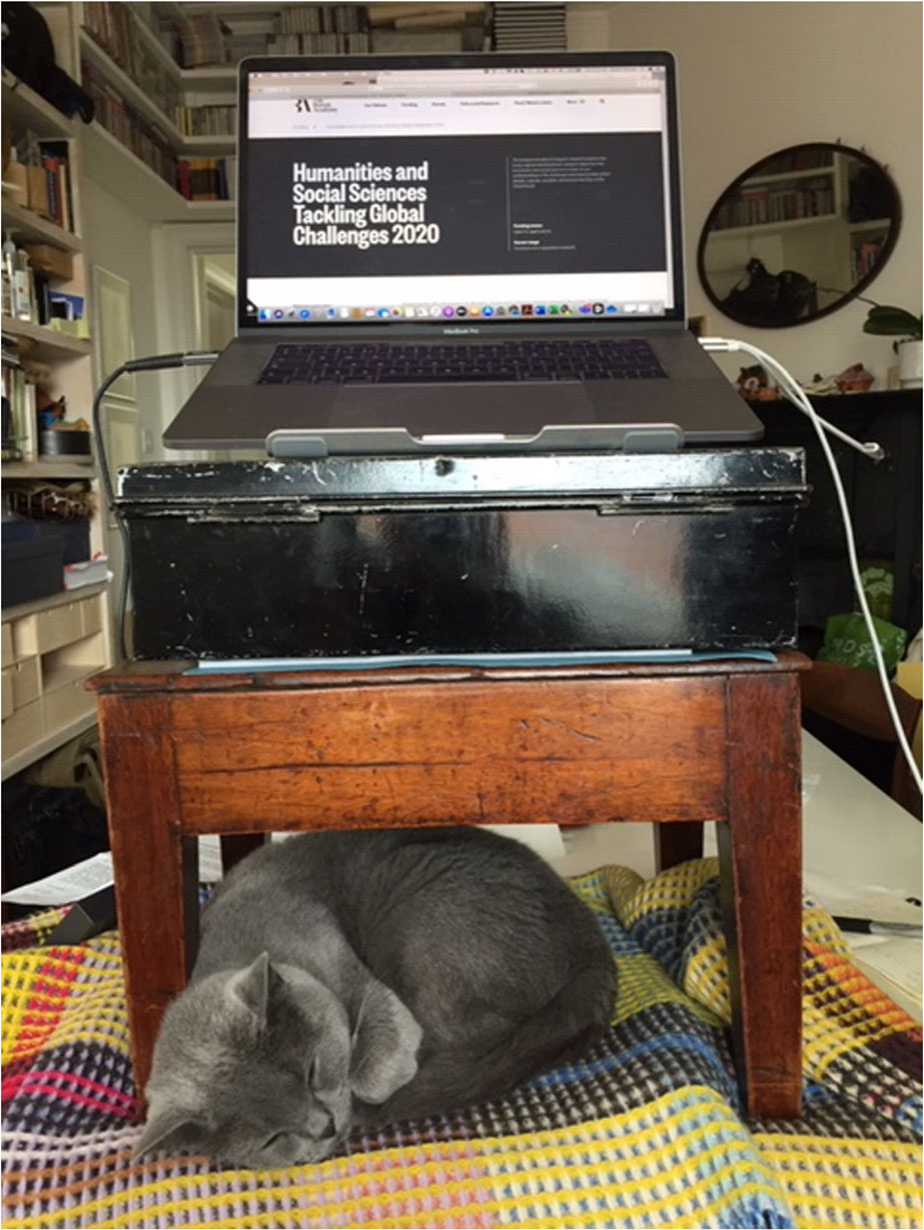
Image provided by Lawrence

Lawrence’s account suggests a sense of being cut off in various ways, both in practical terms and also in terms of community. He sent the image (Fig. 4) via email after his interview.

It shows a table with a blanket on top of it, with another small table on the blanket, and what looks like a case or box on top of that, with his laptop on the top of the ‘tower’. His cat nestles under this construction. Rather like Ellie, Lawrence has engaged in an act of bricolage in order to work. In this case, his striking tower of tables was built as he had an aching neck from looking down at his laptop.

### Mary’s Sewing Machine Table

**Fig. 5:**
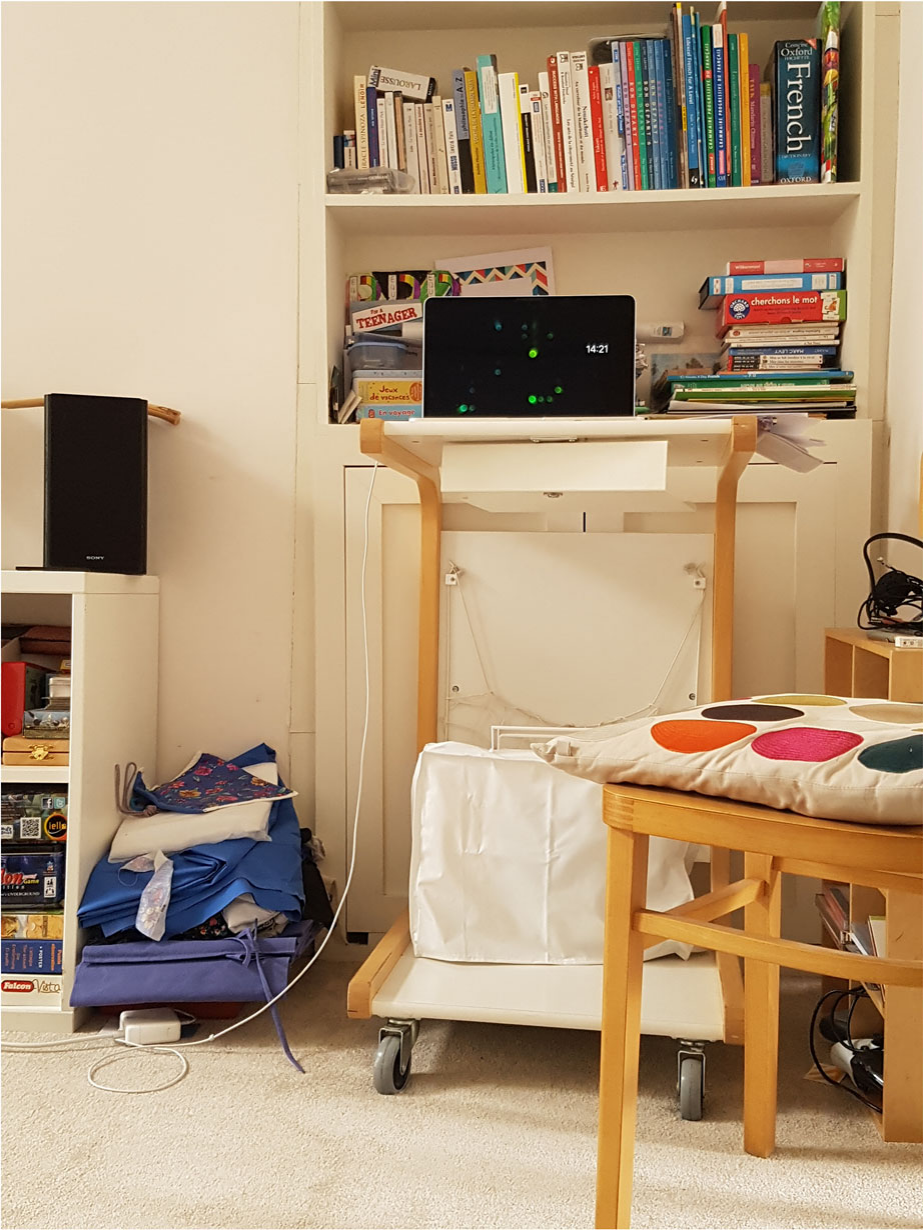
Image provided by Mary

Mary is a Professor in her academic department. I asked Mary about whether it was a challenge to find a work space, and she describes the setup depicted in Fig. 5.

It turns out she has adapted her sewing machine table, which she also uses for making face masks:

Uh, there’s a bedroom, a spare bedroom I use as a study so I can work in there if I need to. I mean, another day I had to spend was cleaning up the study because it was just piled up with papers and stuff [laughter]. So, I, so I cleaned that up. I mean, I often work, you know, anywhere. If I’m reading, I lie on the couch or…Occasionally I’d sit in the kitchen and work, um. And then I’ve set up a little arrangement here with an old, I mean I can show you this (moves laptop to show me the desk and chair in the picture above). I’ve got this old desk. Oh, normally at the bottom is my, I have a sewing machine, which I was making masks with. So, this doubles as my [laughing], my sort of… I think it’s the type of thing for old, you know, when you had a desktop computer really, it was from that stage. But what’s really good is it’s a great height for these meetings.

Mary’s assemblage is of particular interest as the table has a hybrid function which relates directly to the pandemic; she uses the same table to sew face masks. She has adopted the use of the table because of its height, as it is suitable for video calls, not something she required before.

### Elisabetta’s Zoom Call

Elisabetta is a Teaching Fellow. In her interview, she tells me that her elderly father died near the beginning of the lockdown, and she had to return to her home country at short notice. On the day of the funeral, she was asked to arrange her online exam. Her account of that period is one of extreme stress, in a situation where she is providing childcare and home schooling for her two children. She provided an image which consisted of a screenshot of herself and a research collaborator at another university (not shown here for ethical reasons). She describes what had happened that day, and also another incident during a video call:

Because my son has been ill the night before. I’ve been rushing to tidy up the flat and send everyone to their room in order to do this interview…. I remember very well because… In fact, I remember that morning. So, they had said to me, is this day okay? This time okay? And I said, fine, fine. And then I can’t remember what happened. I couldn’t sleep. I think it was when I had my toothache. So, I couldn’t sleep at all in the night, and my toothache was, take… I, I, I had it here. It took my eyes and my brain, and, and I couldn’t let them down because I think it was her but another five people in the background conducting this interview from different universities. And so, um, I remember in some of the questions all I could think of was, I feel so ill. [Laughs]. To be sick [laughing] and, and, and again was, I think, maybe early morning, so, or early morning for my kid. So, for an example, today, I was, uh, having a meeting with the steering committee of the, um, uh, uh, [child’s voice] research board, and I, I was asked a question. And at that moment, my son appeared half naked [laughing]… Shouting something. And I had to concentrate, reply to this question, and this was a perfect occasion in, uh… The meeting had been going on for over an hour, but the moment I was asked a question [laughing]…

Elisabetta’s child was also seeking her attention throughout my interview with her, which was held in what appeared to be the living room of her house. She mentioned that her husband had a home office, but she worked in the communal space. Her account is striking in terms of contrast between the domestic chaos and difficulties she was dealing with at home, in addition to severe toothache, (which she could not have treated because of the lockdown), and her calm and professional demeanour required during the Zoom call.

## Forms of Sequestration

Referring back to the facets of the word *sequestrato* discussed earlier in this paper, the tensions and contradictions inherent in the term seem to emerge in the research participants’ accounts. Courtney describes how her sleeping problems have been resolved during lockdown and states that she has enjoyed the whole experience, especially the absence of commuting. Nuala also reports improved health, despite a heavy workload. In their cases, lockdown seems to have had some of the curative qualities of a period of confinement described by Bashford above; despite the strong differences in their situations, there is a sense of seclusion as a healing experience in their accounts. In contrast, Ellie’s account has a much stronger sense of enforced exile and isolation. She has left London against her will and wants to return to campus as soon as possible. To her, the risk to her mental health is far greater than the risk posed by the virus. Sophia is literally in exile in her own country, after a traumatic journey back. Elisabetta is struggling with domestic demands and stress. Lawrence’s account of his experience also suggests exile and confusion.

## Spatial Assemblage and Bricolage

Most of the participants describe an unfolding ‘making’ process, in order to create some of work space. For some, this involved adding a work ‘layer’ to a space or set of artefacts also used for another purpose. In Lawrence’s case, he constructed his ramshackle structure of tables and boxes in order to be able to work comfortably. (Another participant’s ‘work space’ consisted of her sitting on her toddler’s rocking chair in the nursery room.) Mary used her sewing machine table as a stand for her laptop. Ellie transformed her bedside table into a miniature desk which was in fact too small to hold her laptop and notebook at the same time. Courtney rigged up a curtain to create a ‘professional’ background in her bedroom.

In these cases, a space which is domestic and intimate has been altered, and its fundamental status has been changed. Viewed from a *sociomaterial* perspective as discussed above, these can be regarded as contingent and shifting assemblages of human and nonhuman actors, as opposed to the more conventional view of a work space as a ‘context’, and digital and other technologies as inert ‘tools’ or ‘equipment’. The sudden and enforced nature of the lockdown necessitated this sort of creative improvisation, in which spaces which were hitherto private, domestic, and intimate are change in their nature, arguably becoming outposts of the campus and the world of work.

## Performing the University

A striking theme throughout the data is the central importance of the screen and the video call to the participants. The work space is not primarily focused on ‘study’ in the sense that might be expected from academics, involving reading or writing academic research. The majority of the participants in fact reported that they had been forced to abandon their research work since the beginning of the lockdown, due to the pressures of converting their courses online or in some cases supporting others to do so, in addition to being unable to access field sites and take part in other planned research activities. Instead, they report a great deal of time spent on video calls. This creates a situation in which we can see the contemporary lockdown diverges in many important respects from the states of ‘sequestration’ described by Bashford. The individuals in the study are simultaneously in a state of confinement and separation from the material campus and the physical co-presence of their colleagues and students, but are also required to be in regular or constant contact. The isolation is physical, but not linguistic/social in the sense of communication.

The video call seems central to this ecosystem, and several participants refer to the need to perform a particular type of identity on these calls. Elisabetta’s account of her concealing her toothache and domestic life in order to make a Zoom call with a researcher in another country, Courtney’s ‘professional backdrop’ curtain, and Mary’s enrolling of the sewing machine table for its suitable height for video calls all suggest the screen as a kind of *portal*, through which a professional identity must be performed. In this regard, the situation of these academics under lockdown has an added layer of complexity not apparent in historical spaces of confinement. They are at once physically confined in a domestic space and also required to consistently project a professional identity, outwards via the screen. The site of isolation is therefore permeable, and arguably performative. Their accounts reveal background confusion, overwork, uncertainty, physical disorder, unstable bricolage, domestic mess, noise, unruly children, pets, and also more seriously, profound psychological distress in many cases. But the rectangular portal of their screen frames them as performers, and also, I would argue, as objects of surveillance, who are required—while exiled from the physical campus and its embodied community—to ‘perform the university’, in the context of online teaching this may be analysed as a ‘synthetic situation’ (Knapp 2020). This is a theme which I regard as worthy of further exploration in terms of theoretical and empirical considerations of the nature of remote digital working online in academic contexts, and is explored in more detail in Gourlay (2020).

## Discussion

The novel coronavirus is unable to survive for a long period of time outside of the human body, and in that sense, it can be said to only reside in a vast distributed network of human bodies worldwide. This provokes the question for me as to where then does the university reside, where is it situated? Normally, this question would be regarded as absurd, and the common-sense reply would be ‘the campus’. However, under lockdown, the campus is locked and inaccessible, a space which cannot be entered; it is almost entirely uninhabited. It was abandoned with no warning, its inhabitants forced to leave behind their possessions, spaces, work, artefacts, and crucially their co-present academic communities and personal friends without ceremony. At the height of the lockdown, it was regarded as a place of potential contamination, rather like the plague ships of the early modern period discussed above.

The question arises, is the university something which is now empty, that we have vacated it? Where is it? It still continues as an institution, but it has no functioning physical locus, except crucially for the computer servers which maintain the means for digital contact. It is characterised now by human absence. It might be said to now primarily exist instead in the thousands of locked down bodies; it is scattered, distributed, and atomised. In unseen and also deliberately occluded ways, each of these dispersed human bodies participates in an individual and collective simulation, or approximation, of the university as it should normally be configured. This, as the analysis above suggests, is achieved through an unfolding and intricate range of emergent sociomaterial and embodied micro-entanglements which render the private and domestic space hybrid, complex, and compromised. Collectively, it could be argued, lockdown asks us to create an illusion that the university is somehow still the entity it was, situated as it was. This is attempted by requiring the dispersed and sequestered bodies, many mired in confusion and contingency, to engage in an elaborate performance of wholeness, coherence, organisation, and professionalism via performances in front of the ‘portal’ of a screen.

If we recognise that the site of the university is no longer the campus, and is not replaced by online provision, but is in fact sited in the dispersed bodies of the staff and students, then fundamental shifts are required in how we view the ongoing situation of online teaching and working at home (or ‘living at work’). In a literal sense, I would propose that it is not accurate to talk about ‘online teaching and learning’, as none of the messy, embodied, awkward, emotional, emergent, and difficult sociomaterial activity required for it is anywhere else but in, and entangled with, sequestered human bodies and their nonhuman companions. Instead, an alternative conception of digital engagement as *fundamentally material in its nature* may allow us greater theoretical purchase on the messy realities of remote digital engagement in higher education, for academics, professional services staff, and indeed students, whose practical circumstances are likely to be even more challenging. This shift away from an emphasis on the ‘virtual’ and ‘online learning’ may facilitate a greater sensitivity towards the challenges, both practical and emotional, faced by these bodies, simultaneously dispersed, isolated, and connected in this uncanny contemporary state of Covid-19 *quarantena*.

## Data Availability

Transcripts are held securely by the project team.
